# Discontinuation of tyrosine kinase inhibitors in CML patients in real-world clinical practice at a single institution

**DOI:** 10.1186/s12885-018-5167-y

**Published:** 2018-12-12

**Authors:** Nuno Cerveira, Bruno Loureiro, Susana Bizarro, Cecília Correia, Lurdes Torres, Susana Lisboa, Joana Vieira, Rui Santos, Dulcineia Pereira, Cláudia Moreira, Sérgio Chacim, Nélson Domingues, Ana Espírito-Santo, Isabel Oliveira, Ilídia Moreira, Luísa Viterbo, Ângelo Martins, Manuel R. Teixeira, José M. Mariz

**Affiliations:** 1 Department of Genetics, Portuguese Oncology Institute, Porto, Portugal; 2 Department of Onco-Haematology, Portuguese Oncology Institute, Porto, Portugal; 3 Institute of Biomedical Sciences (ICBAS), University of Porto, Porto, Portugal

**Keywords:** Chronic myeloid leukemia, TKI discontinuation, TKI therapy duration, Sustained deep molecular response, Unsustained deep molecular response

## Abstract

**Background:**

Most patients with chronic myeloid leukemia (CML) treated with tyrosine kinase inhibitors (TKIs) will relapse if treatment is withdrawn, but various trials have recently demonstrated that a significant proportion of patients who achieved a stable and deep molecular response (DMR) can stop therapy without relapsing. However, most information on treatment cessation was obtained from clinical trials with strict recruiting criteria.

**Methods:**

We evaluated the outcome of 25 patients with CML that discontinued TKI therapy in our institute in real-world clinical practice.

**Results:**

Of the 25 patients, 76% discontinued therapy in sustained deep molecular response (SDMR) and 24% were in unsustained DMR (UDMR). Discontinuation of therapy due to adverse effects was observed in 5 and 50% of the patients in the SDMR and UDMR groups, respectively. After TKI discontinuation, patients were followed for a median of 24 months. At the time of this analysis, 56% patients had a molecular relapse after a median of 4 months. SDMR and longer treatment duration were associated with lower probability of molecular relapse: 25% in SDMR patients with TKI treatment > 96 months and 85% in UDMR patients with TKI treatment ≤96 months. All relapsed patients promptly resumed TKI therapy and regained at least major molecular response (MMR).

**Conclusions:**

Our results suggest that TKI discontinuation is safe outside clinical trials and particularly effective in CML patients who are in SDMR with longer TKI treatment duration.

**Electronic supplementary material:**

The online version of this article (10.1186/s12885-018-5167-y) contains supplementary material, which is available to authorized users.

## Background

Chronic myeloid leukemia (CML) is a myeloproliferative neoplasia caused by the fusion of the *BCR* and *ABL1* genes, most frequently as the result of the reciprocal translocation t(9;22)(q34;q11). ABL1 tyrosine kinase inhibitors (TKI) have dramatically improved the outcome for CML patients. Indeed, when TKI therapy is addressed appropriately, it can led to an optimal molecular response in the majority of CML patients and a life expectancy that approaches that of the general population [1, 2].

Although the ELN (European LeukemiaNet) recommendations on the management of CML propose indefinitely continuation of TKI therapy in all responding patients [3], the latest update of the NCCN (National Comprehensive Cancer Network) guidelines [4], based on the most recent studies on treatment-free remission, support the view that treatment can be interrupted in a selected group of patients. However, due to the inability of all TKIs to eliminate quiescent leukemic stem cells, the majority of the patients will require TKI therapy to be continued indefinitely, since most will relapse if treatment is withdrawn [5–7]. Nevertheless, lifelong TKI therapy may have consequences, including chronic, mostly low-grade, adverse events that can substantially impact patients’ quality of life, adherence to therapy and, consequently, success of treatment [8]. In the last few years, several clinical discontinuation trials have demonstrated that 40–60% of chronic phase CML patients (CP-CML) who have achieved a stable deep molecular response (DMR), defined as a sustained molecular response of at least 4.5 (MR^4.5^), can stop therapy without relapsing [reviewed in 9,10]. In addition to DMR, other variables that have been associated with a successful treatment-free remission (TFR) include low Sokal risk group at diagnosis, chronic-phase patients, optimal response to TKI therapy, longer duration of TKI therapy (> 8 years), and longer duration of DMR (> 2 years) [9]. Recently, natural killer (NK) cell number and function at the time of discontinuation were associated with outcome after imatinib discontinuation in chronic-phase CML patients in deep molecular response [10]. In all published trials, the majority of patients who experienced relapse did so within 6 months of TKI cessation and, with the exception of one case that progressed to blast crisis, relapsed patients remained responsive to retreatment and regained at least a major molecular response (MMR) [9,10). However, most information on treatment cessation was obtained from clinical trials with strict recruiting criteria. In this study, we retrospectively aimed to assess persistence of TFR in 25 CML patients treated at our institution that discontinued therapy due to several causes, including DMR and TKI intolerance, and to identify factors that could be associated with TFR.

## Methods

### Patients

The medical records of all CP-CML patients who were treated with TKIs in our institution between 1997 and 2015 were reviewed to identify patients that discontinued TKI therapy due to any reason. This retrospective study was in agreement of our institutional protocol for the management of patients with chronic myeloid leukemia. The eligibility criteria were CP-CML, treated with any of the first-line approved TKIs (imatinib, nilotinib, and dasatinib), and with typical transcripts [that is, b3a2 (e14a2) and/or b2a2 (e13a2)] determined by RT-PCR at the time of diagnosis. Recorded data included patient characteristics at diagnosis, the treatment received prior to discontinuation, the reasons for treatment discontinuation and the course of disease before and after discontinuation. Informed consent was registered in the clinical record of the patients in agreement with the Declaration of Helsinki.

### Cytogenetic and molecular evaluation

Cytogenetic analysis was performed on bone marrow cells using standard G-banding methods on at least 20 metaphases from 24 h cell cultures, at diagnosis and at 3 months intervals during the first year, until a complete cytogenetic response (CCyR) was achieved. Molecular monitoring was performed by reverse transcriptase quantitative PCR (RT-qPCR) with an assay sensitivity of ≥4.5 log and scoring of deep molecular responses (DMR; MR^4.0^, MR^4.5^ and MR^5.0^) was made according to published recommendations [3, 11]. After stopping TKI treatment, patients were, whenever possible, monitored by RT-qPCR monthly during the first year, then every 2 months for the second year, and every 3 months thereafter. Sustained DMR (SDMR) was defined as at least a MR^4.5^ sustained during at least 2 years, and unsustained DMR (UDMR) was defined as at least a MR^4.5^ with less than 2 years. Molecular relapse was defined as loss of major molecular response [MMR; *BCR-ABL1*/*ABL1* internationally standardized (IS) ratio ≤ 0.1%], which triggered TKI resumption.

### Statistical analyses

All statistical analyses were performed using SPSS software (SPSS Inc., Chicago, Ill., USA). Study variables were summarized using standard descriptive statistics and measures of central tendency, including median, ranges, frequencies and percentages. Time to molecular relapse was measured from the date of TKI discontinuation to the date of molecular relapse or the date of last molecular evaluation for patients who did not relapse. Treatment-free survival was estimated using the Kaplan-Meier method. To establish factors associated with TFR, patients with a follow-up of at least 16 months after TKI discontinuation were analyzed. We assessed age, sex, Sokal risk group, *BCR-ABL1* transcript type, duration of TKI therapy, and DMR duration as potential prognostic factors. Differences in molecular relapse between groups were compared using the log-rank test. A two-tailed *p*-value of < 0.05 was considered to indicate statistical significance.

## Results

### Patient demographics and clinical characteristics at diagnosis

Twenty-five patients with chronic phase CML diagnosed and/or treated at our institution agreed to participate in the study and their baseline characteristics are detailed in Table 1. Fifteen patients (60%) were female and median age of all patients was 54 years (range, 15–78 years). Sokal score was low, intermediate and high in 10 (40%), 10 (40%) and 5 (20%) patients, respectively.

**Table 1:** Patient demographics and clinical characteristics at diagnosis

Parameter	Data
Gender	
Male	10 (40%)
Female	15 (60%)
Age (years)	
Median	54
Range	15–78
Sokal score at diagnosis	
Low	10 (40%)
Intermediate	10 (40%)
High	5 (20%)

### Patient characteristics at TKI discontinuation

All patients were in chronic phase and none had a previous history of allogeneic stem cell transplantation (Table 2). Median age at TKI discontinuation was 64 years (range, 24–84). Imatinib as first line therapy was given to 23 (92%) patients, either soon after CML diagnosis (*n* = 20) or after intolerance to IFN-α (*n* = 3). Two patients (8%) received nilotinib as first line therapy. Median duration of TKI treatment before discontinuation was 100 months (range, 25–202) and, with the exception of one patient that was treated for only 25 months, all patients received TKI treatment during at least 36 months. Median time to DMR was 56 months (range, 13–207) in 24 patients, excluding the patient treated for 25 months that failed to achieve DMR. The median duration of DMR before stop was 41 months (range, 0–100). At the time of therapy discontinuation, 20 (80%) patients were receiving their initial TKI (19 imatinib and one nilotinib), four (16%) had changed to a second TKI due to intolerance (three from imatinib to dasatinib and one from nilotinib to imatinib), and one (4%) had changed from imatinib to dasatinib due to resistance.

**Table 2:** Patient characteristics at TKI discontinuation

Parameter	Data
Age, years	
Median	64
Range	24–84
Duration of TKI therapy before stop, months	
Median	100
Range	25–202
Time to DMR, months	
Median	56*
Range	13–207
DMR duration, months	
Median	44
Range	(0–100)
Initial treatment	
IFN-α	3 (12%)
Imatinib	20 (80%)
Nilotinib	2 (8%)
Treatment at discontinuation	
Imatinib	20 (80%)
Nilotinib	1 (4%)
Dasatinib	4 (16%)
Reason for TKI discontinuation	
SDMR	19 (76%)
SDMR	18 (95%)
SDMR/adverse event	1 (5%)
UDMR	6 (24%)
UDMR/clinician and patient decision	2 (33%)
UDMR/adverse event	3 (50%)
UDMR/pulmonary tuberculosis	1 (17%)
Response at TKI discontinuation	
MR^5.0^	12 (48%)´
MR^4.5^	12 (48%)
MR^4.0^	1 (4%)
MMR	0 (0%)

*One patient did not achieved DMR and was not included in this analysis; TKI, tyrosine kinase inhibitor; DMR, deep molecular response; SDMR, sustained deep molecular response; UDMR, unsustained deep molecular response;MR^5.0^, ≥5-log reduction from IRIS baseline; MR^4.5^, ≥4.5-log reduction from IRIS baseline; MR^4.0^, ≥4.0-log reduction from IRIS baseline; MMR, major molecular response

Nineteen patients (76%) interrupted TKI therapy in SDMR and, with the exception of one case that developed pleural effusion (PE) under dasatinib treatment, none presented major adverse events at the time of discontinuation. The remaining six patients interrupted treatment in UDMR: two due to shared clinician and patient decision (both under imatinib treatment), three due to adverse events (one under treatment with dasatinib that developed PE, one under treatment with dasatinib that developed both PE and pulmonary hypertension, and one under treatment with nilotinib that developed acute renal failure) and one that discontinued imatinib due to onset of pulmonary tuberculosis. Molecular response at TKI cessation was MR^5.0^ in 12 patients (48%), MR^4.5^ in 12 patients (48%), and MR^4.0^ in one patient (4%).

### Clinical course after TKI discontinuation

**Fig. 1:**
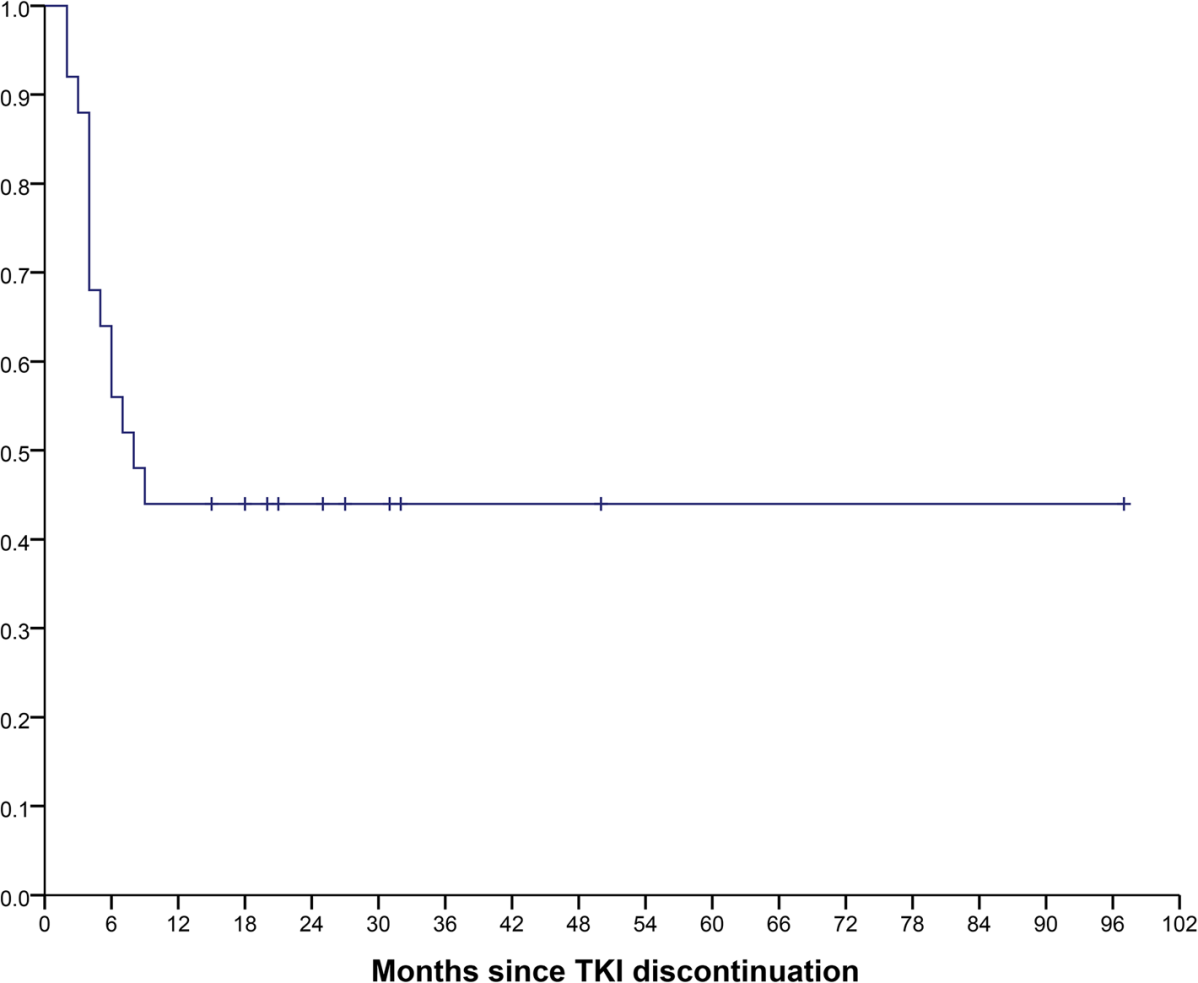
Kaplan-Meier estimates of treatment-free remission after TKI discontinuation in patients with chronic myeloid leukemia. The estimated survival without molecular relapse (SWMR) at 12 months was 45.4% (95% CI 27.5–63.3) for all patients

After TKI discontinuation, patients were followed for a median of 24 months (range, 15–97) (Table 3). At the time of this analysis, 14 patients (56%) had a molecular relapse after a median of four months (range, 2–9). Their characteristics, treatment and response are summarized in Additional file 1: Table S1. The estimated survival without molecular relapse (SWMR) for all patients at 12 months was 45.4% (95% CI 27.5–63.3; Fig. 1). The majority of relapses (79%) occurred within the first six months after TKI discontinuation, with the earliest relapse occurring after two months and the latest at nine months. Relapsed patients promptly resumed TKI therapy, and all regained a MMR after a median duration of TKI treatment of two months (range, 1–10). Of these, at the time of the last evaluation, 10 achieved MR^4.5^ or better, after a median time of five months (range, 2–10) (Table 3, Additional 1: Table S1). None of the two patients with a relapse within two months of TKI discontinuation was in SDMR. The first (patient 3, Additional file 1: Table S1) was treated with imatinib for 10 months and was in DMR for 10 months at the time of TKI suspension. After relapse, imatinib was restarted and the patient was in MMR at the last follow-up. The second (patient 5, Additional file 1: Table S1) was treated for a total of 126 months: first with imatinib for 24 months but, due to treatment intolerance, he was changed to second–line dasatinib. Under dasatinib, this patient developed PE and pulmonary hypertension and, for this reason, a TKI discontinuation was attempted when the patient was in DMR for only eight months. After molecular relapse, imatinib treatment was initiated and the patient achieved a MMR. Patient 6 developed resistance under first-line imatinib after 68 months of treatment. *BCR-ABL1* mutational analysis was negative and the patient was changed to second-line dasatinib. He was treated for an additional 44 months, achieved a DMR and was in SDMR for 33 months at the time of TKI discontinuation. He relapsed after six months, which prompted dasatinib resumption and was in MR^5.0^ at the last evaluation. Of the three patients that received initial treatment with IFN-α before imatinib, one relapsed four months after TKI discontinuation (patient 2, Additional file 1: Table S1). This patient was treated with IFN-α for eight months, followed by imatinib for 171 months and, at the time of TKI discontinuation, was in DMR for only 10 months. At relapse, treatment with dasatinib was instituted and the patient regained a DMR (MR^5.0^). Patient 10 developed acute renal failure under treatment with nilotinib and treatment was interrupted. The patient was not in SDMR and relapsed after seven months. Nilotinib therapy was reinstituted and, at the last follow-up, the patient was in MR^4.0^ with a normalized renal function. Patient 14 was in treatment with imatinib for 25 months and in MR^4.0^ when he acquired pulmonary tuberculosis. TKI treatment was discontinued and the patient relapsed after three months. At this point, imatinib therapy was resumed, with the patient achieving a MR^4.5^ at the last follow-up. Patient 9 was diagnosed with CML outside our institution and first-line therapy with nilotinib was started. After six months, due to patient preference, he was transferred to our institute. Molecular evaluation showed a MR^4.0^ and, according to our institute protocol for first-line treatment of CML patients, he was changed to imatinib. The patient was in SDMR for 56 months when TKI discontinuation was attempted but relapse occurred after four months. Imatinib therapy was resumed and at the last follow-up the patient was in MR^4.0^.

**Table 3:** Clinical course after TKI discontinuation

Parameter	Data
Follow-up after TKI discontinuation (all patients), months	
Median	24
Range	15–97
Molecular relapse	
Total	14 (56.0%)
SDMR	9 (47.4%)
UDMR	5 (83.3%)
Time to molecular relapse, months	
Median	4
Range	2–9
Time to molecular relapse (SDMR patients), months	
Median	5
Range	4–9
Time to molecular relapse (UDMR patients), months	
Median	3
Range	2–7
Time to MMR after molecular relapse, months	
Median	2
Range	1–10
Time to MR ≥ 4.5 after molecular relapse, months	
Median	5
Range	2–10
Response at last follow-up (all patients)	
MR^5.0^	15 (60%)
MR^4.5^	5 (20%)
MR^4.0^	2 (8%)
MMR	3 (12%)

TKI, tyrosine kinase inhibitor; SDMR, sustained deep molecular response; UDMR, unsustained deep molecular response; MR^5.0^, ≥5-log reduction from IRIS baseline; MR^4.5^, ≥4.5-log reduction from IRIS baseline; MR^4.0^, ≥4.0-log reduction from IRIS baseline; MMR, major molecular response

**Fig. 2:**
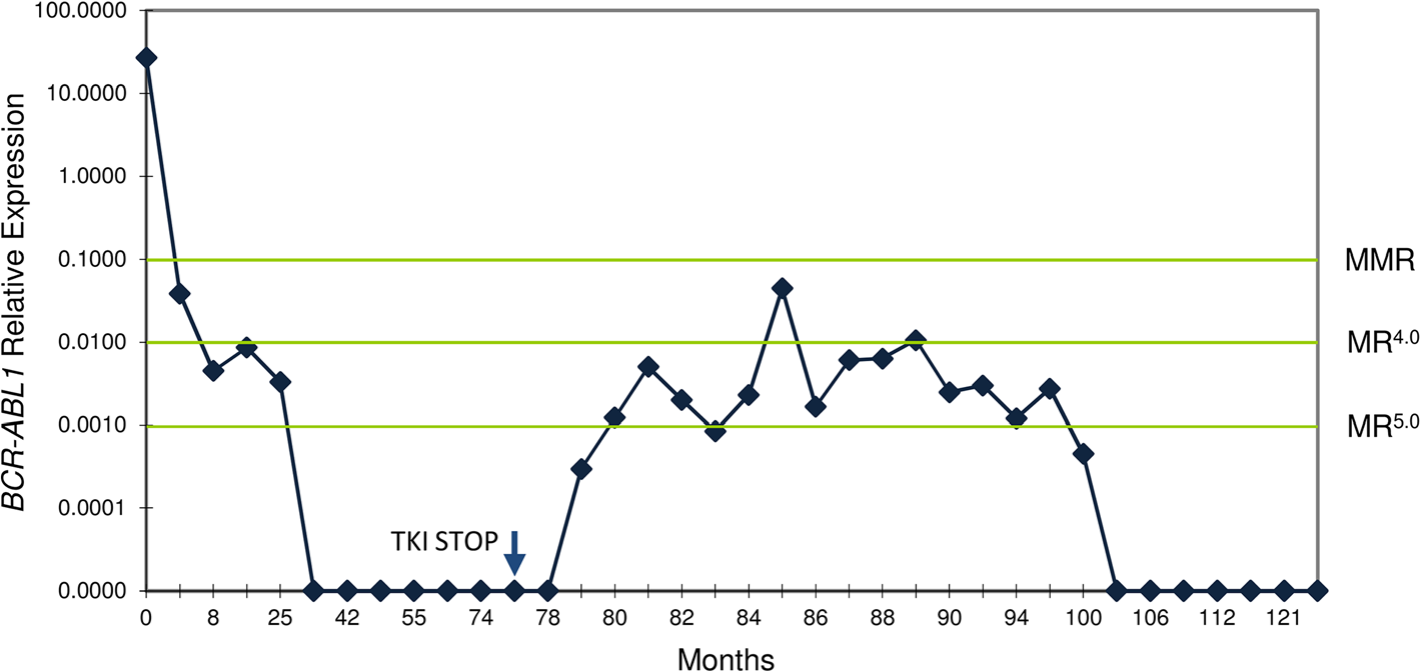
Molecular monitoring of *BCR-ABL1* levels of patient 24 since diagnosis. The patient achieved a DMR after 30 months of imatinib therapy and was in MR^4.5^ (undetectable disease) at the time of TKI discontinuation. After therapy interruption, the patient showed oscillations in *BCR-ABL1* levels until she achieved a MR^5.0^ with undetectable disease 50 months after imatinib discontinuation. In this patient, no therapeutic intervention directed to CML was performed since TKI discontinuation

At the last evaluation, 11 patients (44%) remain treatment-free with a median follow-up from treatment discontinuation of 27 months (range, 15–97), including two patients treated with dasatinib that discontinued therapy due to PE (patients 16 and 17, Additional file 2: Table S2). Of note, patient 16 was previously treated with IFN-α for 36 months but developed intolerance. For that reason, he was switched to imatinib but, due to persistent eosinophilia, was changed to dasatinib. At the time of TKI interruption both patients were in DMR: patient 16 in UDMR for 10 months and patient 17 in SDMR for 41 months and, at the last evaluation, they were relapse-free, after 17 and 27 months of follow-up, respectively. At the data cut-off, eight non-relapsing patients (73%) are in MR^5.0^, two (18%) are in MR^4.5^, and one (9%) is in MMR. In all cases, fluctuations in *BCR-ABL1* levels were observed after treatment discontinuation. Three patients (27%), showed an improvement of their molecular response from therapy discontinuation to the last follow-up (MR^4.5^ to MR^5.0^), and one patient (9%) showed a detriment of their molecular response (MR^4.5^ to MMR). In seven patients (63%), five in MR^5.0^ and two in MR^4.5^, no change in their molecular response level was observed at the last follow-up when compared to the discontinuation date. Of note, patient 24 showed a particularly interesting pattern of variation of *BCR-ABL1* levels after TKI discontinuation (Additional file 2: Table S2 and Fig. 2). This patient was a female treated for 77 months with imatinib who achieved a DMR after 30 months and was in SDMR (MR^4.5^, undetectable disease) at the time of TKI discontinuation. Five months later, increasing *BCR-ABL1* levels lead to loss of MR^4.5^ (*BCR-ABL1* = 0.0050%; MR^4.0^) but, subsequently declining *BCR-ABL1* levels resulted in a MR^5.0^ (*BCR-ABL1* = 0.0008%) seven months after TKI interruption. This was again followed by increasing levels of *BCR-ABL1*, leading to loss of DMR to a MMR (*BCR-ABL1* = 0.0447%) at the 9th month evaluation. She regained DMR (MR^4.5^) at 10 months but again, increasing transcript levels, resulted in a MMR (*BCR-ABL1* = 0.0106%) 13 months after TKI discontinuation. In the following months she showed a decreasing trend of *BCR-ABL1* levels, with acquisition of DMR and, at the last follow-up, 50 months after imatinib discontinuation, she is in MR^5.0^ with undetectable disease. In this patient, no therapeutic intervention directed to CML disease was performed since TKI discontinuation.

### Parameters associated with survival without molecular relapse

Statistical analysis was performed to identify factors associated with survival without molecular relapse (Table 4). In our series, the molecular relapse rate was 47.4% (nine patients) in SDMR patients, after a median of five months (range, 4–9). Most of the relapses occurred within the first six months (79%), with two patients relapsing at eight and nine months, respectively. In this group, the estimated SWMR was 53.7% (95% CI 33.1–74.2). Five out of six patients (83.3%) in the UDMR group relapsed after a median of three months (range, 2–7), with an estimated SWMR of 6.3% (95% CI 1.3–11.4). The only patient in the UDMR group that did not relapse interrupted therapy as a result of PE. Also in this group most of the relapses (80%) occurred in the first six months. The probability of SWMR was statistically different between patients in SDMR when compared with patients in UDMR (*p* = 0.016, Table 4). SWMR was not only significantly associated with SDMR but also with the duration of TKI therapy. Indeed, therapy duration time > 60 months (*p* = 0.026) and > 96 months (*p* = 0.040) before treatment interruption was significantly associated with the probability of molecular relapse: 45 and 100% in the group of patients treated with TKI > 60 months or ≤ 60 months, and 38 and 89% in the group of patients with TKI > 96 months or ≤ 96 months, respectively. The estimated SWMR at 12 months was 55.4% (95% CI 35.2–75.6) and 5.4% (95% CI 3.6–7.2) in the group of patients treated with TKI > 60 months or ≤ 60 months, and 62.0% (95% CI 39.9–84.1) and 10.7% (95% CI 1.5–19.8) in the group of patients with TKI > 96 months or ≤ 96 months, respectively.

**Table 4:** Potential factors associated with treatment-free remission

Parameter	Patients	Estimated SWMR at 12 months, % (95% CI)	*p* value
Age, years			0.965
> 64	12	16.6 (9.1–24.0)	
≤ 64	13	47.0 (21.8–72.2)	
Sex			0.325
Male	10	59.6 (31.2–88.0)	
Female	15	20.3 (9.6–30.9)	
Sokal risk score			0.707
Low	10	59.5 (31.0–88.0)	
Intermediate	10	13.4 (6.5–20.3)	
High	5	9.4 (5.5–13.3)	
Transcript type			0.393
e14a2	14	51.5 (27.7–75.3)	
e13a2	7	15.3 (5.2–25.4)	
e14a2 + e13a2	4	8.3 (1.0–15.5)	
TKI therapy, months			0.026
> 60	20	55.4 (35.2–75.6)	
≤ 60	5	5.4 (3.6–7.2)	
TKI therapy, months			0.040
> 96	16	62.0 (39.9–84.1)	
≤ 96	9	10.7 (1.5–19.8)	
Molecular Response			0.016
SDMR	19	53.7 (33.1–74.2)	
UDMR	6	6.3 (1.3–11.4)	
Molecular Response + TKI therapy, months			0.003
SDMR + > 60	16	62.7 (41.0–84.4)	
UDMR + ≤60	9	6.1 (2.7–9.6)	
Molecular Response + TKI therapy, months			0.003
SDMR + > 96	12	73.9 (51.3–96.5)	
UDMR + ≤96	13	11.9 (2.9–20.8)	

TKI, tyrosine kinase inhibitor; SDMR, sustained deep molecular response; UDMR, unsustained deep molecular response

**Fig. 3:**
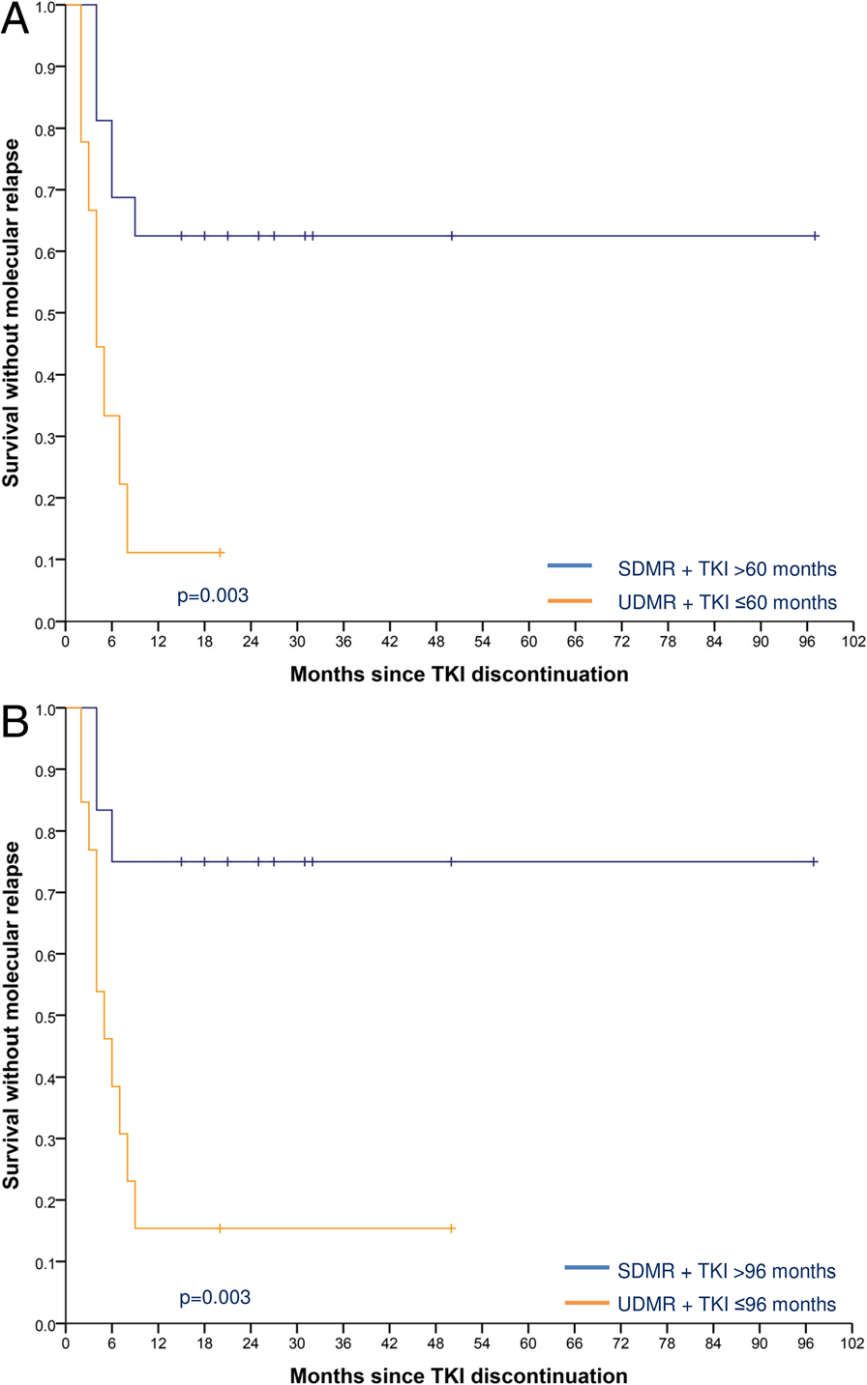
Kaplan-Meier estimates of treatment-free remission after TKI discontinuation in patients with chronic myeloid leukemia. (A) The estimated survival without molecular relapse (SWMR) at 12 months was 62.9% (95% CI 41.1–84.4) in SDMR patients with TKI treatment > 60 months when compared with only 6.1% (95% CI 2.7–9.6) in UDMR patients with TKI treatment ≤60 months. (B) The estimated survival without molecular relapse (SWMR) at 12 months was 73.9% (95% CI 51.3–96.5) in SDMR patients with TKI treatment > 96 months compared with 11.9% (95% CI 2.9–20.8) in UDMR patients with TKI treatment ≤96 months

When both the depth of molecular response and TKI therapy duration were considered, the probability of molecular relapse was 37.5% in SDMR patients with TKI treatment > 60 months compared to 88.9% in UDMR patients with TKI treatment ≤60 months (*p* = 0.003), with an estimated SWMR at 12 months of 62.7% (95% CI 41.1–84.4) in the former compared with only 6.1% (95% CI 2.7–9.6) in the later (Fig. 3a). With longer treatment duration, the probability of molecular relapse was 25.0% in SDMR patients with TKI treatment > 96 months patients and 84.6% in UDMR patients with TKI treatment ≤96 months (p = 0.003). The estimated SWMR at 12 months was 73.9% (95% CI 51.3–96.5) in SDMR patients with TKI treatment > 96 months compared with 11.9% (95% CI 2.9–20.8) in UDMR patients with TKI treatment ≤96 months (Fig. 3b).

We did not find any statistically significant association between SWMR with age, gender, Sokal score, and transcript type.

## Discussion

In the present study, we evaluated the outcomes of CP-CML patients who discontinued TKI therapy in our institution due to any reason including SDMR and/or adverse events. Our data show that 44% of the patients remained in TFR at 12 months, with an estimated SWMR of 45.4%, a value lower than that observed in clinical trials that used MMR loss as a trigger to restart TKI therapy, which is around 50–60% [12–14]. This may be explained by the fact that our series includes patients that discontinued TKI therapy for any reason, namely patients without SDMR, whose molecular relapse rate was 83%, with an estimated SWMR of only 5.0%. If we use more strict criteria, such as patients in SDMR at the time of therapy discontinuation, the TFR rate rises to 53%, more in line with the results observed in clinical trials. These results support the hypothesis that a sustained deep molecular response, defined as a MR^4.5^ for at least 2 years, is associated with a greater probability of TFR. Using this criterion, the proportion of imatinib-treated CML patients eligible to a TFR attempt is approximately 30–40% after 5 years of therapy [15]. However, using more potent TKIs, such as nilotinib and dasatinib, the rates can raise up to 42–54% [16, 17]. The rates seem to slowly but steadily increase over time, raising the possibility that with very prolonged treatment the level of minimal residual disease may continue to fall. This can in turn increase the pool of CML patients that can access treatment discontinuation. However, it is a matter of intense debate whether a sustained MR^4.5^ should be a strict requirement for TKI discontinuation, with preliminary results from the EURO-SKI trial suggesting that it could be safe to stop treatment in patients in MR^4.0^, and that TKI treatment duration could be more important than the deepness of molecular response [18]. In previous clinical trials, imatinib treatment duration has emerged as a continuous variable, with increasing length of therapy correlated to increased success of TFR [13, 19, 20]. Supporting this hypothesis, we also found that a duration of TKI therapy both, over 60 months (5 years) and 96 months (8 years), was significantly associated with a lower probability of molecular relapse after TKI discontinuation: 45 and 38% in the group of patients treated with TKI > 60 months and TKI > 96 months, respectively. In addition, when both SDMR and longer TKI treatment duration were used as TFR selection criteria, the rates of molecular relapse were even lower: 37.5 and 88.9% in SDMR patients treated with TKI > 60 months or ≤ 60 months, and 25.0, and 84.6% in SDMR patients treated with TKI > 96 months or ≤ 96 months, respectively. Our data suggest that both the duration of DMR and time on TKI treatment should be carefully evaluated when TFR is considered. Importantly, it is not known if attempting TFR after a shorter duration of TKI treatment may reduce the likelihood of a successful outcome and eventually compromise the long-term success of TFR.

In addition to the degree of molecular response and treatment duration, several studies have suggested that other patient characteristics, including age, gender and Sokal score, could be associated with successful TFR, but results have not been consistent between studies [18, 21]. In our series, we did not find any statistically significant association between TFR with age, gender, and Sokal score. Furthermore, it has been suggested that the *BCR-ABL1* transcript type could predict for optimal ELN responses and for longer event-free and transformation-free survival [22]. Therefore, we also evaluated the potential impact of transcript type (e14a2, e13a2 and e13a2 + e14a2) on TFR, but again no association could be found. We nevertheless recognize that much larger series are needed to conclusively evaluate other variables besides DMR and treatment duration.

In our series, five out of six patients that interrupted therapy in UDMR relapsed. The reasons for discontinuation included personal option (two cases), intolerance or toxicity issues (three cases) and pulmonary tuberculosis (one case), which clearly illustrates that following strict criteria in the selection of patients eligible for drug discontinuation is not always feasible outside clinical trials. Three patients under treatment with dasatinib discontinued therapy due to PE. Interestingly, two of them remain free of treatment, one that interrupted treatment in SDMR and the other in UDMR. PE is a common side-effect of dasatinib treatment with an incidence ranging from 14 to 39% [16, 23]. Although, most cases are mild or moderate, with grade 3/4 reported in only 3% of patients, PE represents one of the leading causes of dasatinib discontinuation [16, 23, 24]. It has been suggested that an inflammatory immune response involving NK cells underlies the development of PE in patients receiving dasatinib, a mechanism that could be associated with tumor regression [25, 26]. Interestingly, evidence implicating the role of immune response in TFR is increasing. The abundance and activity of NK cells, T-regulatory cells and dendritic cells has been correlated with likelihood of TFR [27–29], with recent data suggesting that NK cells may play a role controlling leukemia-initiating cells responsible for relapse after TKI cessation [10]. This can explain fluctuations in *BCR-ABL1* transcript levels below the level of MMR, observed in most of our patients that remain in TFR. This phenomenon implies persistence of molecular disease due to a residual pool of leukemia stem cells (LSC) and that NK cells might contribute to prevent overt CML relapse [7]. These data highlight a potential role of the immune system in controlling residual LSCs, which may not be BCR-ABL1 addicted and therefore may be resistant to TKI treatment and represent a potential risk of relapse.

In our series, most relapses occurred early in the first 6 months after TKI discontinuation and late relapses were a rare event, which is in agreement with most of the studies published to date [9, 30]. All our relapsed patients responded to TKI treatment and regained at least a MMR with no progression to advanced phase, also in accordance to previously published data [9, 30]. This seems to support the hypothesis that the TFR period does not favor the emergence of resistant clones, as long as TKI treatment is reintroduced soon after molecular relapse is identified. Only two exceptions to this scenario were reported in the literature: in one patient MMR loss was accompanied by the detection of a nilotinib-resistant F359 V mutation, whereas in another patient, who was in MMR after imatinib resumption, CML progressed to lymphoid blast crisis [12, 31]. Indeed, it would be surprising if TFR was completely innocuous, with a very small risk of subsequent progression impossible to exclude.

Despite the excitement over TKI discontinuation, only a minority of newly diagnosed CML patients are likely to enter successful TFR, assuming relapse is defined as loss of MMR. For the remaining majority, lifelong TKI therapy may be required. An important practical question is when to consider TFR outside clinical trials. Our data, reflecting “real-world” clinical practice without selection of patients, support the hypothesis that patients in long-term TKI treatment and sustained deep molecular response are clearly the best candidates. However, access to high quality internationally standardized qRT-PCR, rapid turn-around of qRT-PCR test results, and structured follow-up established to enable rapid intervention in cases of molecular relapse are also mandatory.

## Conclusions

Despite the small and heterogeneous group of patients, our results are in agreement with recent published data regarding TKI discontinuation success in patients with CML. We here show that TKI discontinuation is feasible and safe outside clinical trials, being particularly effective in CML patients in SDMR and with longer duration of TKI treatment.

## Additional files

Additional file 1: **Table S1.** Outcome of CML patients who restarted TKI treatment after molecular relapse. (DOCX 16 kb)

Additional file 2: **Table S2.** Outcome of CML patients who remain in treatment-free remission. (DOCX 16 kb)

## Abbreviations

CCyR: Complete cytogenetic response
CML: Chronic myeloid leukemia
CP-CML: Chronic phase chronic myeloid leukemia
DMR: Deep molecular response
ELN: European LeukemiaNet
MMR: Major molecular response
NCCN: National Comprehensive Cancer Network
NK: Natural killer
PE: Pleural effusion
SDMR: Sustained deep molecular response
SWMR: Survival without molecular relapse
TFR: Treatment-free remission
TKI: Tyrosine kinase inhibitor
UDMR: Unsustained deep molecular response
