# Supplementary material for: Discontinuation of tyrosine kinase inhibitors in CML patients in real-world clinical practice at a single institution
Source: BMC Cancer. 2018 Dec 12;18:1245. doi: 10.1186/s12885-018-5167-y (PMC6292043; doi:10.1186/s12885-018-5167-y)
Supplement: Supplementary file 1 — Table S1. Outcome of CML patients who restarted TKI treatment after molecular relapse. (DOCX 16 kb) [file 12885_2018_5167_MOESM1_ESM.docx]

**Additional Table 1 – Outcome of CML patients who restarted TKI treatment after molecular relapse**

| Patient | Age range at Diagnosis  (years) | Sokal | EUTOS | Transcript Type | TKI Treatment Duration | DMR duration | Response at STOP | TKI at STOP | STOP  Duration  (months) | TKI  Restarted | Time to Response  (months) | Response at Last Follow-Up |
| --- | --- | --- | --- | --- | --- | --- | --- | --- | --- | --- | --- | --- |
| 1 | 50-59 | Int | Low | b2a2 | 74 | 25 | MR^4.5^ | Imatinib | 6 | Imatinib | 1 | MR^5.0^ |
| 2 | 40-49 | Int | NA | b3a2 | 171 | 10 | MR^5.0^ | Imatinib | 4 | Dasatinib | 1 | MR^5.0^ |
| 3 | 40-49 | Low | Low | b2a2 | 140 | 10 | MR^4.5^ | Imatinib | 2 | Imatinib | 3 | MMR |
| 4 | 70-79 | Int | Low | b3a2 | 108 | 29 | MR^4.5^ | Imatinib | 4 | Imatinib | 3 | MR^5.0^ |
| 5 | 60-69 | Low | Low | b2a2 | 126 | 8 | MR^4.5^ | Dasatinib | 2 | Imatinib | 10 | MMR |
| 6 | 40-49 | Low | NA | b3a2 | 112 | 33 | MR^5.0^ | Dasatinib | 6 | Dasatinib | 1 | MR^5.0^ |
| 7 | 50-59 | Int | Low | b3a2 | 98 | 46 | MR^5.0^ | Imatinib | 4 | Imatinib | 3 | MR^5.0^ |
| 8 | 70-79 | High | Low | b3a2 | 85 | 45 | MR^5.0^ | Imatinib | 9 | Imatinib | 1 | MR^5.0^ |
| 9 | 50-59 | Int | NA | b2a2/b3a2 | 71 | 56 | MR^5.0^ | Imatinib | 4 | Imatinib | 5 | MR^4.0^ |
| 10 | 70-79 | High | Low | b3a2 | 42 | 22 | MR^4.5^ | Nilotinib | 7 | Nilotinib | 1 | MR^4.0^ |
| 11 | 60-69 | High | Low | b2a2/b3a2 | 60 | 33 | MR^5.0^ | Imatinib | 5 | Imatinib | 1 | MR^4.5^ |
| 12 | 70-79 | High | High | b3a2 | 49 | 24 | MR^4.5^ | Imatinib | 8 | Imatinib | 3 | MR^4.5^ |
| 13 | 30-39 | Int | Low | b2a2 | 37 | 24 | MR^5.0^ | Imatinib | 4 | Imatinib | 1 | MR^5.0^ |
| 14 | 40-49 | Low | Low | b2a2/b3a2 | 25 | 0 | MR^4.0^ | Imatinib | 3 | Imatinib | 4 | MR^4.5^ |

TKI, Tyrosine Kinase Inhibitor; Int, Intermediate; DMR, Deep Molecular response; MR^5.0^, ≥5-log reduction from IRIS baseline; MR^4.5^, ≥4.5-log reduction from IRIS baseline; MR^4.0^, ≥5-log reduction from IRIS baseline; MMR, major molecular response.
