# Supplementary material for: Discontinuation of tyrosine kinase inhibitors in CML patients in real-world clinical practice at a single institution
Source: BMC Cancer. 2018 Dec 12;18:1245. doi: 10.1186/s12885-018-5167-y (PMC6292043; doi:10.1186/s12885-018-5167-y)
Supplement: Supplementary file 2 — Table S2. Outcome of CML patients who remain in treatment-free remission. (DOCX 16 kb) [file 12885_2018_5167_MOESM2_ESM.docx]

**Additional Table 2 – Outcome of CML patients who remain in treatment-free remission**

| Patient | Age range at Diagnosis  (years) | Sokal | EUTOS | Transcript Type | TKI Treatment Duration | DMR duration | Response at STOP | TKI at STOP | STOP  Duration  (months) | Best Response | Worst Response | Response at Last Follow-Up |
| --- | --- | --- | --- | --- | --- | --- | --- | --- | --- | --- | --- | --- |
| 15 | 40-49 | Low | Low | b3a2 | 100 | 74 | MR^4.5^ | Imatinib | 97 | MR^5.0^ | MR^4.5^ | MR^5.0^ |
| 16 | 50-59 | Low | Low | b2a2 | 180 | 10 | MR^4.5^ | Dasatinib | 20 | MR^4.0^ | MMR | MMR |
| 17 | 30-39 | Int | High | b2a2 | 202 | 41 | MR^4.5^ | Dasatinib | 27 | MR^5.0^ | MR^4.5^ | MR^5.0^ |
| 18 | 50-59 | Int | Low | b3a2 | 144 | 56 | MR^4.5^ | Imatinib | 15 | MR^5.0^ | MMR | MR^4.5^ |
| 19 | 70-79 | Int | Low | b2a2 | 153 | 100 | MR^4.5^ | Imatinib | 25 | MR^5.0^ | MR^4.5^ | MR^5.0^ |
| 20 | 50-59 | Low | Low | b3a2 | 143 | 48 | MR^5.0^ | Imatinib | 32 | MR^5.0^ | MR^4.0^ | MR^5.0^ |
| 21 | 50-59 | Low | Low | b3a2 | 116 | 56 | MR^5.0^ | Imatinib | 50 | MR^5.0^ | MR^4.0^ | MR^5.0^ |
| 22 | 60-69 | Int | Low | b2a2/b3a2 | 136 | 60 | MR^5.0^ | Imatinib | 21 | MR^5.0^ | MR^4.5^ | MR^5.0^ |
| 23 | 50-59 | Low | Low | b2a2 | 99 | 84 | MR^5.0^ | Imatinib | 32 | MR^5.0^ | MR^4.5^ | MR^5.0^ |
| 24 | 30-39 | Low | Low | b3a2 | 77 | 47 | MR^4.5^ | Imatinib | 50 | MR^5.0^ | MMR | MR^5.0^ |
| 25 | 10-19 | High | Low | b3a2 | 97 | 59 | MR^5.0^ | Imatinib | 21 | MR^5.0^ | MR^4.5^ | MR^5.0^ |

TKI, Tyrosine Kinase Inhibitor; Int, Intermediate; DMR, Deep Molecular response; MR^5.0^, ≥5-log reduction from IRIS baseline; MR^4.5^, ≥4.5-log reduction from IRIS baseline; MR^4.0^, ≥5-log reduction from IRIS baseline; MMR, major molecular response.
